# The clinical use of remote parameter testing during cardiac implantable electronic devices implantation procedures: a single center, randomized, open-label, non-inferiority trial

**DOI:** 10.3389/fcvm.2024.1364940

**Published:** 2024-03-22

**Authors:** Shiqiang Xiong, Shujuan Qin, Lin Tong, Yu Long, Yan Luo, Qiao Feng, Xiufen Peng, Maoling Jiang, Feng Xiong, Jin Li, Yangchun Zhang, Zhen Zhang, Hanxiong Liu, Lin Cai

**Affiliations:** Department of Cardiology, The Third People’s Hospital of Chengdu, Affiliated Hospital of Southwest Jiaotong University, Chengdu Cardiovascular Disease Research Institute, Chengdu, Sichuan, China

**Keywords:** cardiac implantable electronic devices, remote testing, remote interrogation, remote reprogramming, implantation, COVID-19

## Abstract

**Background:**

A novel non-contact system for remote parameter testing and reprogramming offers an alternative method for assessing device parameters during cardiac implantable electronic devices (CIEDs) implantation without the need for physical contact with the manufacturer's clinical service technician. The safety and feasibility of using this system in CIEDs implantation procedures remains to be determined.

**Objective:**

Evaluate the safety and feasibility of remote parameter testing in CIEDs implantation procedures.

**Methods:**

A single center, randomized, open-label, non-inferiority trial (ChiCTR2200057587) was conducted to compare the two approaches for interrogating CIEDs during implantation procedures: routine interrogation performed by on-site technicians or remote interrogation performed by technicians using the 5G-Cloud Technology Platform. Patients aged ≥18 years and elected to receive CIEDs were eligible for inclusion. The primary endpoint was the completion rate of the parameter test. Safety and efficiency were evaluated in all randomly assigned participants.

**Results:**

A total of 480 patients were finally enrolled and were randomly assigned to routine group (*n* = 240) or remote group (*n* = 240). The primary endpoint was achieved by 100% in both groups (*P* = 0.0060 for noninferiority). The parameters of sensing, threshold, and impedance regarding the right atrium, right ventricle, and left ventricle had no statistical significance between the two groups (*P* > 0.05). Procedure time, parameter testing time, and both duration and dose of x-ray irradiation were not significantly different between the two groups (*P* > 0.05). Shut-open door frequency was significantly higher in the routine group than the remote group [6.00 (4.00, 8.00) vs. 0, *P* < 0.0001]. Notably, no clinical or technical complications were observed in the remote group.

**Conclusions:**

Remote parameter testing is safe and feasible across various devices implantation procedures. The utilization of remote parameter testing and reprogramming could represent an innovative approach to improve healthcare accessibility and unlock the full potential of secondary centers in managing CIEDs.

**The Registration Identification:**

ChiCTR2200057587.

## Introduction

Cardiac implantable electronic devices (CIEDs) are the most effective means of treating and diagnosing several types of arrhythmias and heart failure (1). To ensure the safety and efficacy, clinicians must evaluate the parameters of the device during the procedure. During routine CIEDs implantation procedures, parameter testing and reprogramming are performed by the manufacturer's clinical service technician in the catheterization laboratory while physically present with the patient. This process inevitably exposes the technician to x-ray radiation, which can potentially cause harmful health effects such as carcinogenesis, gene mutation, and cataracts. Due to the outbreak of the COVID-19 pandemic, the manufacturer's clinical service technicians were banned from entering the catheterization laboratory and ward as a precautionary measure to reduce the potential risk of cross-infection. Meanwhile, the rates of CIEDs implantations declined significantly, especially during the early pandemic wave (2–6). Resolving these issues of parameter testing and programming is absolutely imperative.

A novel real-time system for remote parameter testing and reprogramming, based on China Telecom's 5G-Cloud Technology Platform (5G-CTP), offers an alternative method for assessing device parameters during CIEDs implantation without the need for physical contact with the manufacturer's clinical service technician (7). Recently, the use of this novel noncontact system during CIEDs implantation procedures has been reported in a small, non-randomized study against the background of the global COVID-19 pandemic (8). The significance of this system in clinical practice warrants further investigation. Therefore, the aim of this study was to evaluate the feasibility and safety of using this system in CIEDs implantation procedures through a randomized controlled trial.

## Methods

### Study design

This single center, randomized, open-label study (ChiCTR2200057587) was conducted at the Third People's Hospital of Chengdu (Sichuan Province, China) comparing two approaches for interrogating and reprogramming CIEDs during implantation procedures: routine interrogation and reprogramming performed by on-site technicians (Routine group) or remote interrogation and reprogramming performed by technicians using the 5G-Cloud Technology Platform (Remote group). The study was approved by the institutional review board committee (CSY-2022-S-11) and adhered to Helsinki Declaration guidelines.

The indications for CIEDs implantation were evaluated based on temporal guidelines. Patients were eligible for inclusion if: they were ≥18 years, elected to receive CIEDs manufactured by Abbott (Chicago, Illinois, USA), and provided written informed consent. A total of 480 patients between April 2022 and May 2023 were enrolled and were randomly assigned to either the Routine or Remote group in a 1:1 ratio.

### Remote interrogation and reprogramming solution

**Figure 1:**
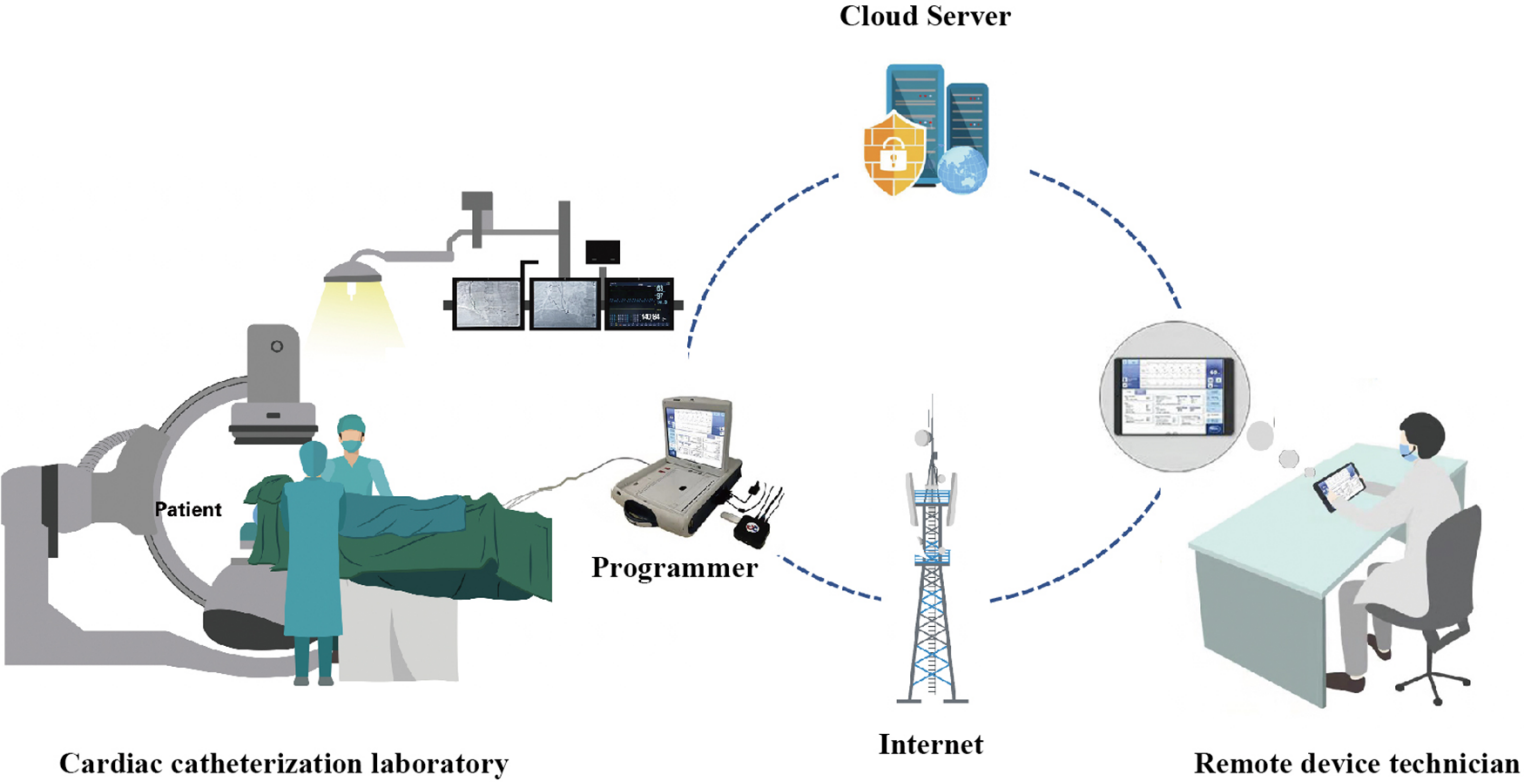
An overview of the utilization of the 5G-cloud technology platform for remote parameter testing. The 5G-cloud technology platform for remote parameter testing, consisting of a 5G remote support terminal connected to the programmer externally, a PAD equipped with a 5G-cloud follow-up application, and a cloud-based remote service system. The 5G remote support terminal connects directly to the 5G-cloud follow-up application via the internet. No network or software is required for the on-site programmer. Remote control of the on-site programmer can be realized by simply connecting to the 5G remote support terminal and using simulated mouse and keyboard information. No direct data interact between the computer and the on-site programmer. This system enables clinical device technicians to provide real-time remote parameter testing and reprogramming for cardiac implantable electronic devices in primary medical and healthcare institutions lacking device specialists.

The 5G-CTP consists of three primary components. First, an off-site device with 5G cloud follow-up software (China Telecom Corporation Limited Shanghai Branch, Shanghai, China). Second, a Merlin Patient Care System Programmer Model 3650 (St. Jude Medical Inc., Minnesota, USA), which is externally connected to the 5G data transmit module (China Telecom Corporation Limited Shanghai Branch, Shanghai, China) in the catheterization laboratory by USB cables. Both surface electrocardiograph and intracardiac signals are displayed on the programmer. Third, a remote service system, which is deployed on cloud servers (China Telecom Cloud, China). The 5G-CTP is a research tool that enables technicians to remotely test and reprogram CIEDs in real-time without the need for physical presence with the patient through an internet connection or mobile wireless network (Figure 1).

The 5G-CTP has implemented specific security procedures to ensure patient safety and cybersecurity. Firstly, an authorized technician must undergo a two-step verification process to log into the 5G-cloud follow-up application. Step 1 requires logging into a designated account with a password, while Step 2 involves using an access password for second verification to establish a remote connection for the designated device. Secondly, it employs a 2048-bit Rivest-Shamir-Adleman asymmetric key exchange, autonomous peer-to-peer data transfer protocol, and anti-cracking measures, building on the advanced encryption standard encryption mechanism to safeguard communication and patient security. Thirdly, the servers are deployed in server rooms equipped with multilayer firewalls, customized antivirus scanning, vulnerability scanning, and intrusion detection to ensure data security. Fourthly, the entire remote operation process is saved through screen recording generation, which allows auditors to review the logs later on. Fifthly, in the event of a communication interruption between the on-site programmer and the remote technician's device, the CIEDs will revert back to the original settings. Furthermore, real-time video and audio communication between technicians and onsite medical staff enhances the security of remote parameter testing and programming. Before commencing the research, all enrolled technicians underwent specialized training for this protocol.

### Data collection

The data on patient's sociodemographic characteristics, medical history, smoking status, laboratory examination, medical information, and procedural information were obtained from the electronic medical records. Medical history data included a history of conditions such as coronary artery disease, stroke, atrial fibrillation, diabetes mellitus, hypertension, dyslipidemia, chronic obstructive pulmonary disease, and chronic renal dysfunction.

The primary hypothesis was that remote interrogation and reprogramming solution is non-inferior to routine methods in achieving freedom from primary endpoints, with a 5% noninferiority margin. Following indices related to CIEDs implantation procedures were gathered: (1) the completion rate of the parameter test; (2) pacing threshold; (3) sensing amplitude; (4) lead impedance; (5) the proportions of the above parameters in the recommended range; (6) duration of intraoperative testing; (7) duration of operation; (8) duration of x-ray irradiation that device specialists received during the testing process; (9) number of door openings related to parameter testing. Pacing parameters were recommended as follows (sensing amplitude/threshold/impedance): right atrium lead: >2.00 mV/<1.50 V/300–1,500 Ω; right ventricular lead: >5.00 mV/<1.00 V/300–1,500 Ω; left ventricular lead: >5.00 mV/<2.00 V/300–1,500 Ω.

### Statistical analyses

It was estimated that 121 patients per study group are needed to support the primary hypothesis with a power of 80% and α level of 0.05. Continuous data were expressed as either mean ± standard deviation (SD) or median [interquartile range (IQR)] for normally or skewed distributed variables, respectively. Statistical analysis between groups employed either Student's t-test or Mann–Whitney *U*-test. Normality of distribution was determined via Kolmogorov–Smirnov and Shapiro–Wilk tests. Categorical variables were presented as numbers (percentages) and compared using the chi-squared test or Fisher's exact test, as appropriate. A two-tailed *P* value < 0.05 was considered significant. All statistical analyses were conducted using SPSS software (version 26.0, SPSS, Inc., Chicago, IL, USA) and Prism version 8.0 (GraphPad software, San Diego, CA, USA).

## Results

A total of 480 patients were finally enrolled and were randomly assigned to routine group (*n* = 240) or remote group (*n* = 240). Baseline characteristics of the individuals stratified by randomization group are shown in Table 1. The primary endpoint was achieved by 100% in both groups (*P* = 0.0060 for noninferiority). The parameters of sensing, threshold, and impedance regarding the right atrium, right ventricle, and left ventricle had no statistical significance between the two groups (Table 2, all *P* > 0.05). The parameter values of the two groups were all within the recommended ranges.

**Table 1:** Patient characteristics at enrollment.

Characteristic	Routine group (*n* = 240)	Remote group (*n* = 240)	*P* value
Sex			0.7839
Female, *n* (%)	111 (46.25)	115 (47.92)	
Male, *n* (%)	129 (53.75)	125 (52.08)	
Age, years	73.00 (65.00, 80.75)	75.00 (68.00, 82.00)	0.0240
BMI, kg/m^2^	23.50 ± 3.41	24.08 ± 3.55	0.0763
SBP, mmHg	132 ± 21.15	136.50 ± 21.23	0.0215
Heart rate, bpm	64.05 ± 18.00	63.02 ± 17.80	0.5278
CIED type, *n* (%)			0.9114
Single-chamber pacemaker.	20	26	
Double-chamber pacemaker	188	182	
Single-chamber ICD	10	10	
Double-chamber ICD	6	7	
CRT-P/D	16	15	
Baseline left ventricle ejection fraction, %	59.99 ± 8.39	60.65 ± 7.79	<0.0001
Coronary artery disease, *n* (%)	55 (22.00)	59 (24.58)	0.5223
Previous stroke, *n* (%)	13 (5.42)	20 (8.33)	0.2790
Arterial fibrosis, *n* (%)	64 (26.67)	70 (29.17)	0.6110
Diabetes, *n* (%)	64 (26.67)	74 (31.62)	0.2661
Hypertension, *n* (%)	144 (60.00)	157 (65.42)	0.2573
Dyslipidemia	48 (20.00)	53 (22.08)	0.6543
COPD, *n* (%)	10 (4.17)	8 (3.33)	0.8110
Chronic renal dysfunction, *n* (%)	27 (11.25)	27 (11.25)	>0.9999
Hemoglobin, g/L	128.80 ± 19.95	129.50 ± 18.68	0.7123
Albumin, g/L	39.38 ± 4.56	39.15 ± 3.83	0.5651
cTnT, pg/ml	18.96 (10.70, 34.38)	14.71 (10.29, 24.96)	0.0144
Uric Acid, μmol/L	385.70 (308.20, 465.50)	369.3 (310.50, 456.20)	0.2140
Scr, μmol/L	83.15 (68.30, 101)	79.05 (64.30, 95.03)	0.0557
BNP, pg/ml	192.30 (77.20, 599.80)	126.90 (56.60, 333.20)	0.0008
Plasma glucose, mmol/L	5.77 (5.01, 7.11)	5.78 (5.05, 7.15)	0.9969
Triglyceride, mmol/L	1.20 (0.88, 1.81)	1.21 (0.93, 1.65)	0.7362
Total Cholesterol, mmol/L	4.25 (3.44, 4.96)	4.19 (3.39, 5.07)	0.9517
HDL-C, mmol/L	1.26 (1.05, 1.49)	1.30 (1.09, 1.57)	0.1401
LDL-C, mmol/L	2.29 (1.81, 2.81)	2.25 (1.69, 2.86)	0.5993

Data are presented as *n* (%), median (IQR) or mean ± standard. BMI, body mass index; SBP, systolic blood pressure; CIED, cardiac implantable electronic device; ICD, implantable cardioverter defibrillator; CRT-P/D, cardiac resynchronization therapy-pacemaker/defibrillator; AVB, atrioventricular block; COPD, chronic obstructive pulmonary disease; Scr, serum creatinine; cTnT, cardiac troponin T; BNP, brain natriuretic peptide; HDL-C, high-density lipoprotein cholesterol; LDL-C, low-density lipoprotein cholesterol.

**Table 2:** Remote parameters testing of CIEDs.

Variables	Routine group (*n* = 240)	Remote group (*n* = 240)	*P* value
Sensing, mV			
Right atrium	2.80 (1.95, 3.80)	2.60 (2.00, 3.70)	0.7871
Right ventricle	11.10 (7.90, 16.35)	11.70 (8.10, 16.10)	0.9548
Left ventricle	11.15 (7.25, 17.83)	10.75 (6.58, 18.00)	0.9714
Threshold, V			
Right atrium	0.70 (0.60, 0.90)	0.70 (0.60, 1.00)	0.2444
Right ventricle	0.60 (0.50, 0.70)	0.60 (0.50, 0.70)	0.7924
Left ventricle	1.00 (0.75, 1.13)	1.00 (0.50, 1.50)	0.5900
Impedance, Ω			
Right atrium	463 (424, 530)	456 (415, 503)	0.1152
Right ventricle	628.50 (558.80, 735.80)	633.50 (568, 709)	0.7600
Left ventricle	694.60 ± 192.60	726.80 ± 241.70	0.7147

Data are presented as median (IQR) or mean ± standard.

Shut-open door frequency was significantly higher in the routine group than the remote group [6.00 (4.00, 8.00) vs. 0, *P* < 0.0001]. Procedure time, parameter testing time, and both duration and dose of x-ray irradiation were not significantly different between the two groups (Table 3, *P* > 0.05). Notably, no clinical or technical complications were observed in the remote group. These results indicate that remote parameter testing is a safe method that does not compromise the efficiency of parameter testing during CIEDs implantation procedures.

**Table 3:** Procedure informations.

Variables	Routine group (*n* = 240)	Remote group (*n* = 240)	*P* value
Shut-open door frequency, *n*	6.00 (4.00, 8.00)	0	<0.0001
Procedure time, min	102 (85.00, 127.00)	100.0 (86.0, 120.0)	0.5187
Parameter testing time, min	39.00 (23.75, 95.00)	35.50 (25.00, 86.50)	0.6401
Parameter testing frequency, *n*	6.00 (4.00, 8.00)	7.00 (5.00, 8.00)	0.0039
x-ray time, s	551.00 (350.80, 813.00)	505.00 (363.30, 723.00)	0.5122
x-ray dose, mGy	19.00 (11.00, 36.00)	18.00 (12.00, 36.25)	0.5122

Data are presented as median (IQR) or mean ± standard.

## Discussion

In this randomized, open-label trial, we found that utilizing remote parameter testing with 5G-CTP during CIEDs implantation procedures is feasible and safe across diverse clinical devices. This system holds the potential to decrease personnel contact, lower the risk of infections like COVID-19, and improve healthcare accessibility.

The sudden outbreak of the COVID-19 pandemic had a profound impact on clinical practice worldwide. The COVID-19 pandemic led to significant declines in the rates of CIEDs implantations during its initial phase. The decrease amounted to 56.50% in northeastern Spain (5), above 40% in England (9), 39.38% in Poland (10), 48% in northwestern Greece (11), 28% in the Veneto region of Italy (12), and 22.90% in Germany (3). The rates of CIEDs implantations also decreased significantly in China ranging from 15.04% to 61.49% between January and May 2020 (2). Possible reasons for the decline in the number of CIEDs implantations in the early stages are as follows: (1) The lack of experience in managing COVID-19 has led to a significant strain on healthcare resources as a result of the sudden surge in cases. Consequently, elective procedures and noncritical visits may be postponed or canceled to conserve hospital resources. (2) Governments have enacted comprehensive public health measures, such as lockdowns and stay-at-home orders, resulting in a reduction in regular medical consultations and subsequently a decline in CIEDs implantations. (3) Many patients, apprehensive about contracting the virus, may choose to forgo seeking medical services, even for severe symptoms. This fear of infection likely contributes to the decrease in CIEDs implantations. (4) To reduce the risk of cross-infection, numerous medical institutions have prohibited the access of manufacturer's clinical service technicians to the catheterization laboratory and ward.

During the COVID-19 pandemic, the utilization of telemedicine has been encouraged in order to minimize unnecessary exposure and its adoption has significantly increased (13). Remote parameter testing, facilitated by 5G-CTP, provides an alternative approach for evaluating device parameters during the implantation of CIEDs, without the need for physical contact with the manufacturer's clinical service technician. Stauning et al. found that the number of airborne colonies was strongly correlated with shut-open door frequency and the number of people present in the operating room (14). During a routine CIED implantation procedure, the manufacturer's clinical service technician is not consistently present in the catheterization laboratory but instead enters when parameter testing and reprogramming are necessary. This practice may lead to more frequent door openings, potentially raising the risk of intraoperative airborne colonies and microbial air contamination, thereby increasing the likelihood of pocket infections. Our study findings demonstrate that implementing this innovative non-contact strategy successfully decreases personnel contact, thereby minimizing the potential risks of cross-infection. This approach maintains the safety and efficacy of remote parameter testing, which is comparable to the standard method. Importantly, utilizing remote parameter testing guarantees the successful completion of CIEDs implantation procedures without compromising patient care. Additionally, clinical service technicians can now circumvent unnecessary exposure to x-ray.

A significant proportion of patients with CIEDs benefit from remote monitoring, which serves as an effective tool for managing cardiac rhythm off-site. According to current guidelines, remote monitoring is highly recommended (class I) for routine use in CIEDs patients (1, 15). However, there is a substantial underutilization of remote monitoring due to various patient-related and system-related challenges. In the context of the pandemic, remote monitoring has been strongly recommended in most circumstances to reduce nonurgent clinic visits (13). The 5G-CTP utilized in the present study not only encompasses real-time remote parameters testing but also incorporates the functionality for remote reprogramming. Therefore, the principles of remote parameter testing can be cautiously extended to the management of CIEDs follow-up, transcending geographic, social, and cultural barriers. This extension is crucial to ensure the continuity of care for CIEDs patients.

Recently, one of our previous studies found that the overall compliance with in-office visits in a single region of China was only 60.60%, highlighting a substantial need for improvement (16). Furthermore, remote monitoring remains significantly underutilized in China due to the absence of reimbursement and logistical support necessary for the effective implementation of closed-loop management. In fact, less than 10% of patients with permanent pacemakers are currently enrolled in remote monitoring services for their devices (17, 18). The security and feasibility of remote reprogramming based on 5G-CTP have been confirmed in our previous series of studies (16, 19–21). Moreover, Mariani et al. demonstrated that virtual visits were equally feasible and effective as in-person consultations, achieving high patient satisfaction among clinical electrophysiology patients amid the COVID-19 pandemic (22). Thus, we propose that remote follow-up based on 5G-CTP may be a novel service model to enhance the management of CIEDs follow-up in patients residing in underserved remote areas (7). Establishing a timely and stable connection between major regional medical centers and surrounding secondary hospitals is of great clinical significance in improving the capacity of secondary medical centers and alleviating the burden on major centers in the implantation and follow-up management of CIEDs. Utilizing 5G-CTP for remote parameter testing and reprogramming may be recommended to unlock the complete capabilities of secondary centers in the management of CIEDs.

### Limitations

This study has some limitations. First, this is a single center, randomized, open-label study. The limitations in generalizability and sample size must be considered when interpreting our findings. Second, our study was limited by the small number of patients implemented with ICD or CRT devices. The CIEDs employed in this study primarily consist of standard pacemakers, representing a notable deviation from the cohort in Western countries. A multi-center, multi-population clinical study is necessary to assess the feasibility of remote parameter testing in CIEDs implantation. Third, we did not conduct follow-up, it remains uncertain whether this non-contact method has the potential to positively impact the clinical prognosis of patients with CIEDs, particularly in terms of pocket infection. Forth, the 5G-CTP is currently compatible only with Abbott (St. Jude) devices. Further exploration to extend this service model to other brands of CIED would yield significant clinical implications. Although certain limitations have been observed, to the best of our knowledge, this is the first randomized controlled trial to validate the safety and efficacy of remote parameter testing in CIEDs implantation.

## Conclusions

Utilizing 5G-CTP for remote parameter testing is safe and feasible across various devices implantation procedures. The principles of remote parameter testing can also be cautiously extended to the management of CIEDs follow-up, offering an innovative approach to enhancing healthcare accessibility and unlock the full potential of secondary centers in managing CIEDs.

## Data Availability

The raw data supporting the conclusions of this article will be made available by the authors, without undue reservation.
